# Effects of Intensive Blood Pressure Reduction on Acute Intracerebral Hemorrhage: A Systematic Review and Meta-analysis

**DOI:** 10.1038/s41598-017-10892-z

**Published:** 2017-09-06

**Authors:** Shun Gong, Chao Lin, Danfeng Zhang, Xiangyi Kong, Jigang Chen, Chunhui Wang, Zhenxing Li, Rongbin Chen, Ping Sheng, Yan Dong, Lijun Hou

**Affiliations:** 1 Department of Neurosurgery, Shanghai Institute of Neurosurgery, PLA Institute of Neurosurgery, Shanghai Changzheng Hospital, Second Military Medical University, 415 Fengyang Road, Shanghai, 200003 China; 2 Department of Radiology, Brigham and Women’s Hospital, Harvard Medical School, 1249 Boylston St., Boston, Massachusetts 02215 United States of America; 3 Department of Neurosurgery, the First Affiliated Hospital of Nanjing Medical University, 300 Guangzhou Road, Nanjing, Jiangsu 210029 China; 4 Department of Neurosurgery, Peking Union Medical College Hospital, Chinese Academy of Medical Sciences, No. 1 Shuaifuyuan Hutong, Dongcheng District, Beijing, 100730 China; 5 Department of Anesthesia, Critical Care and Pain Medicine, Massachusetts General Hospital, Harvard Medical School, Harvard University, 55 Fruit Street, Boston, Massachusetts 02114-3117 United States of America

## Abstract

Current opinions about the effect of intensive blood pressure (BP) reduction for acute intracerebral hemorrhage (ICH) are inconsistent. We performed a meta-analysis to evaluate the efficacy and safety of intensive BP reduction for acute ICH by analyzing data from several recent randomized controlled trials (RCTs). There were six eligible studies that met the inclusion criteria, for a total of 4,385 acute ICH patients in this meta-analysis. After analyzing these data, we found differences between intensive and standard BP lowering treatment groups in total mortality rates, unfavorable outcomes, hematoma expansion, neurologic deterioration, and severe hypotension were not significant. Moreover, compared with the standard treatment, the rate of renal adverse event in intensive treatment group was significantly higher. The intensive treatment approach was recommended in the following situations: (1) longer prehospital duration; (2) lower National Institute of Health stroke scale (NIHSS) score; (3) no hypertension history.

## Introduction

Intracerebral hemorrhage (ICH) accounts for 10–15% of all strokes around the world every year^1^. ICH has been an important public health problem worldwide, with approximately a 50% case fatality at 30 days and high morbidity in survivors^1–3^. The acute hypertensive response in ICH is highly prevalent and is an important prognostic factor^4^. To date, management of blood pressure (BP) has been proven to be safe, but remains uncertain as a method for improving clinical outcomes^5–7^.

The American Heart Association (AHA) previously suggested a target systolic BP of 140 to 179 mm Hg for patients with acute ICH^3^; reducing systolic BP to 140 mm Hg was currently recommended based on a large randomized controlled trial (RCT)^8^. Several RCTs have attempted to illustrate the effects of intensive reduction of elevated BP on the outcomes of patients with ICH^9–12^. Although intensive BP reduction, (defined as a target systolic BP of 110 to 139 mm Hg), did not result in a lower rate of disability or death compared with an standard treatment (defined as a target systolic BP of 140 to 179 mm Hg). Intensive BP reduction has been demonstrated to be associated with beneficial effects of reducing hematoma growth and improving functional outcomes^10, 11^. Other studies and meta-analyses also revealed that intensive BP lowering was safe and might have the potential to improve outcomes and reduce hematoma expansion, but these findings did not reach statistical significance^12–16^. Hence, there is a dispute over how to guide the choice of a target systolic BP when treating acute hypertensive response in patients with ICH.

Therefore, the purpose of this manuscript is to perform a comprehensive systematic review and meta-analysis to evaluate the efficacy and safety of intensive BP lowering compared with standard BP lowering.

## Results

### Description of the selection process

**Figure 1:**
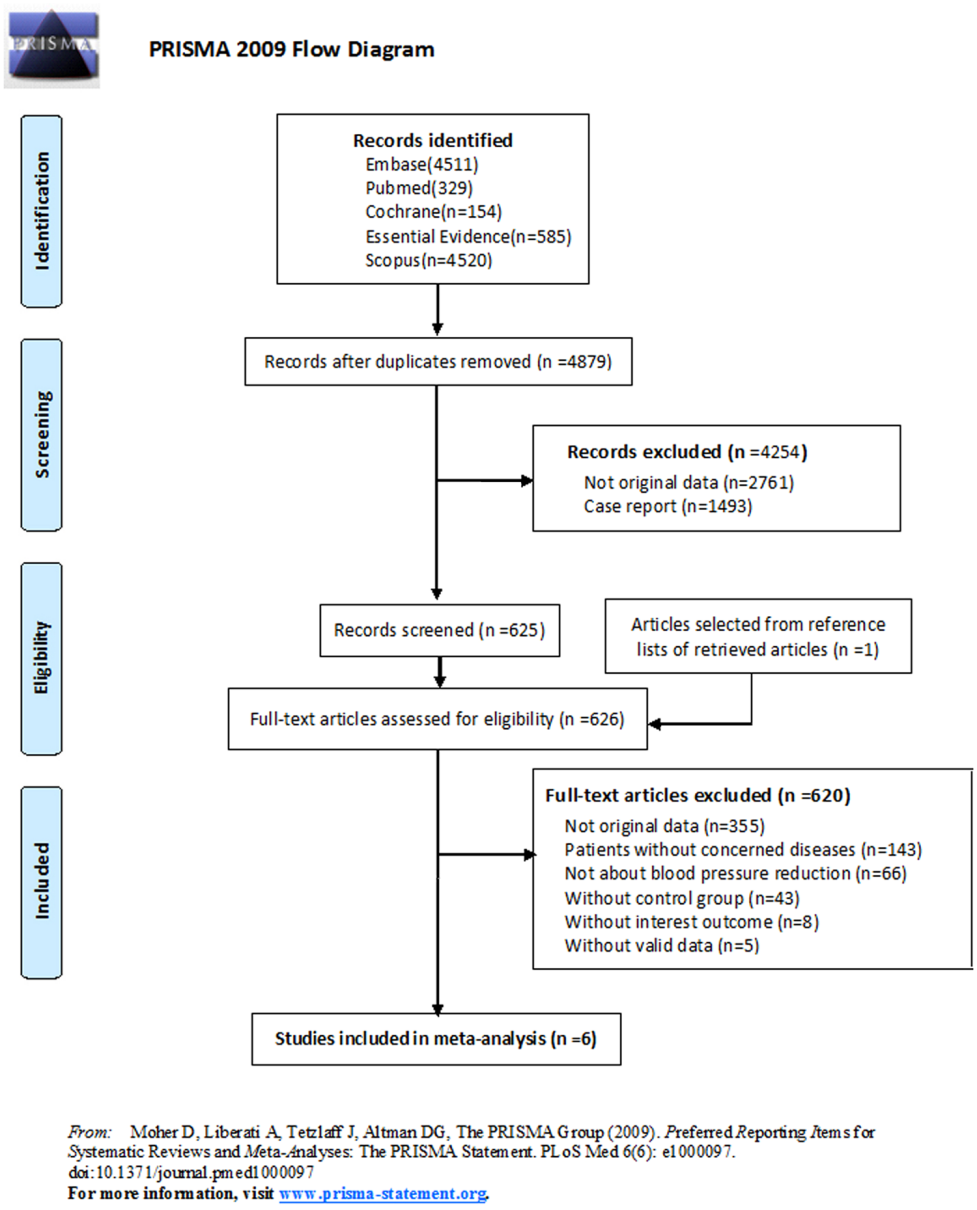
Preferred reporting items for systematic reviews and meta-analyses 2009 flow diagram.

An initial search produced 4,511 results in Embase, 329 results in Pubmed, 154 results in Cochrane, 585 results in Essential Evidence, and 4,520 results in Scopus. There were 4,879 results left after removing the duplicates. We excluded 2,761 results without original data and 1,493 results of case report through analysis of the abstracts. One additional article was selected from the reference lists of retrieved articles. The remaining 626 full-text articles were assessed for eligibility through an in-depth reading of the full text content of each article. Articles not meeting inclusion criteria were excluded: 355 articles without original data, 143 articles without concerned diseases, 66 articles not about BP reduction, 43 articles without control group, 8 articles without interest outcome, and 5 articles without valid data. The final 6 remaining articles are included in this meta-analysis (Fig. 1)^9–12, 17, 18^.

### Description of included studies

**Figure 2:**
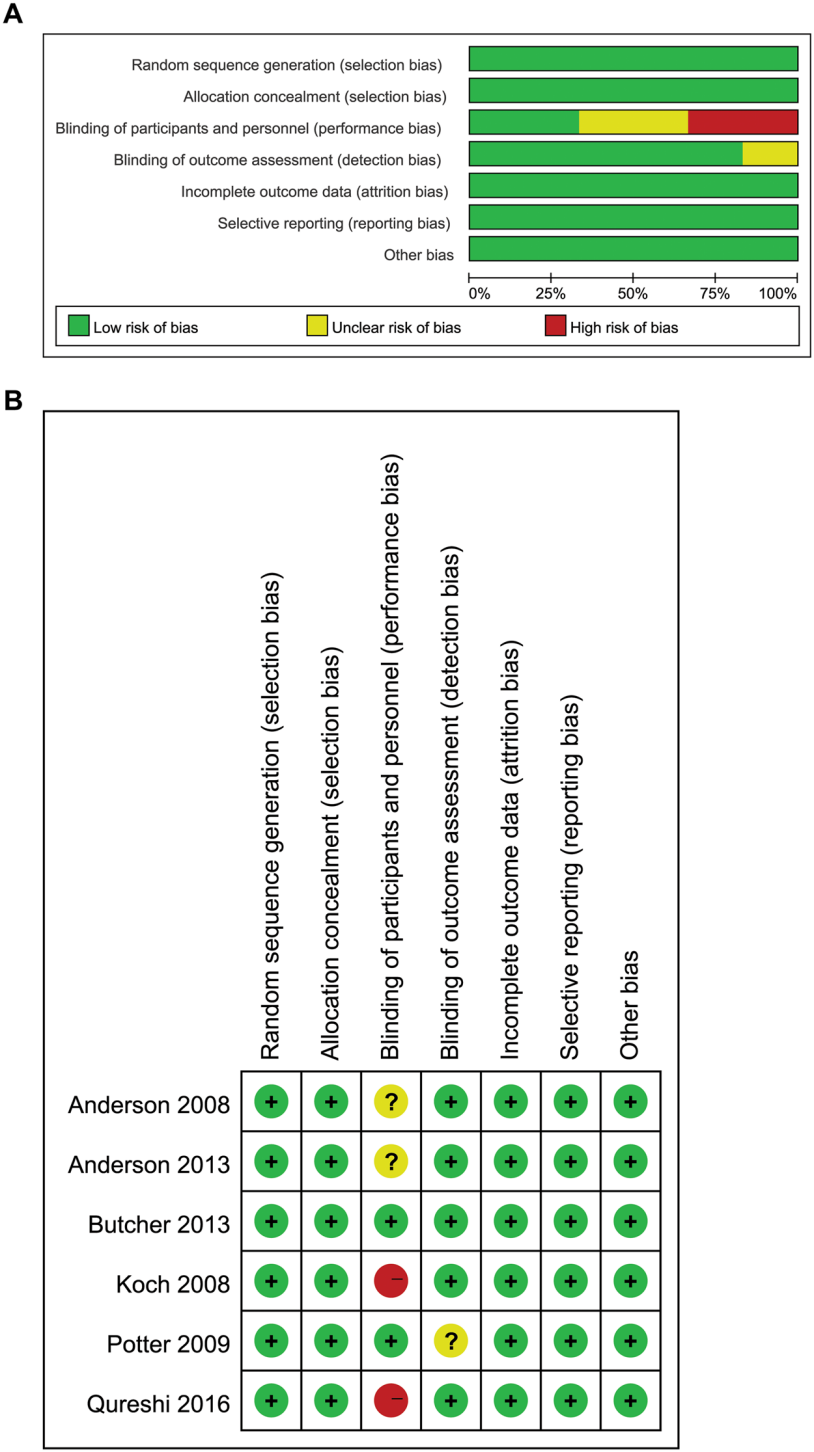
(**A**) Risk of bias graph; (**B**) Risk of bias summary.

Sample sizes of these 6 studies ranged from 25 to 2,839 subjects, with a total of 4,385 subjects. Subject mean age was 63.2 years, and were 62.9% male. Other characteristics of the included studies are summarized in Table 1. Risk of bias of included studies is available in Fig. 2.

**Table 1:** Design and patient characteristics for studies included in the meta-analysis.

Source	Sample size (Intensive/Standard)	Hematoma volume (ml) (Intensive/Standard)	Sex (male)	Mean age (year)	Duration of fellow-up (month)	Baseline BP (mmHg) (Intensive/Standard)	BP target in intensive group (mmHg)	BP target in standard group (mmHg)	Outcomes	Country	Jadad score
Anderson *et al*., 2008	203/201	14.2 ± 14.5/12.7 ± 11.6	64.9%	62.5	3	SBP: 180/182	SBP < 140	SBP < 180	Death or dependency, mRS, NIHSS, Barthel index, AE	Australia	4
Anderson *et al*., 2013	1403/1436	11/11	62.9%	63.5	3	SBP: 179/179	SBP < 140	SBP < 180	Death or dependency, mRS, AE, quality of life	Australia	5
Butcher *et al*., 2013	39/36	25.6 ± 30.84/26.9 ± 25.24	72%	69.7	3	SBP: 182/184	SBP < 150	SBP: 150–180	Mortality, mRS, Barthel Index	Canada	3
Koch *et al*., 2008	21/21	12.5 ± 17.2/8.5 ± 9.8	54.8%	60.6	3	MAP: 144/151	MAP < 110	MAP: 110–130	Mortality, mRS	USA	3
Potter *et al*., 2009	18/7	—	55.2%	74	3	SBP: 182/181	SBP: 145–155	SBP > 155	Dead or dependent, mRS, AE	UK	3
Qureshi *et al*., 2016	500/500	10.3/10.2	62%	61.9	3	SBP: 200/201	SBP: 110–139	SBP: 140–179	Death or dependency, mRS, AE, EQ-5D, NIHSS	USA	5

Abbreviations: AE: adverse event; mRS: Modified Rankin Scale; NIHSS: National Institute of Health stroke scale; BP: blood pressure; SBP: systolic blood pressure; MAP: mean arterial pressure; EQ-5D: European Quality of Life-5Dimensions.

### Primary outcomes

#### Mortality

**Figure 3:**
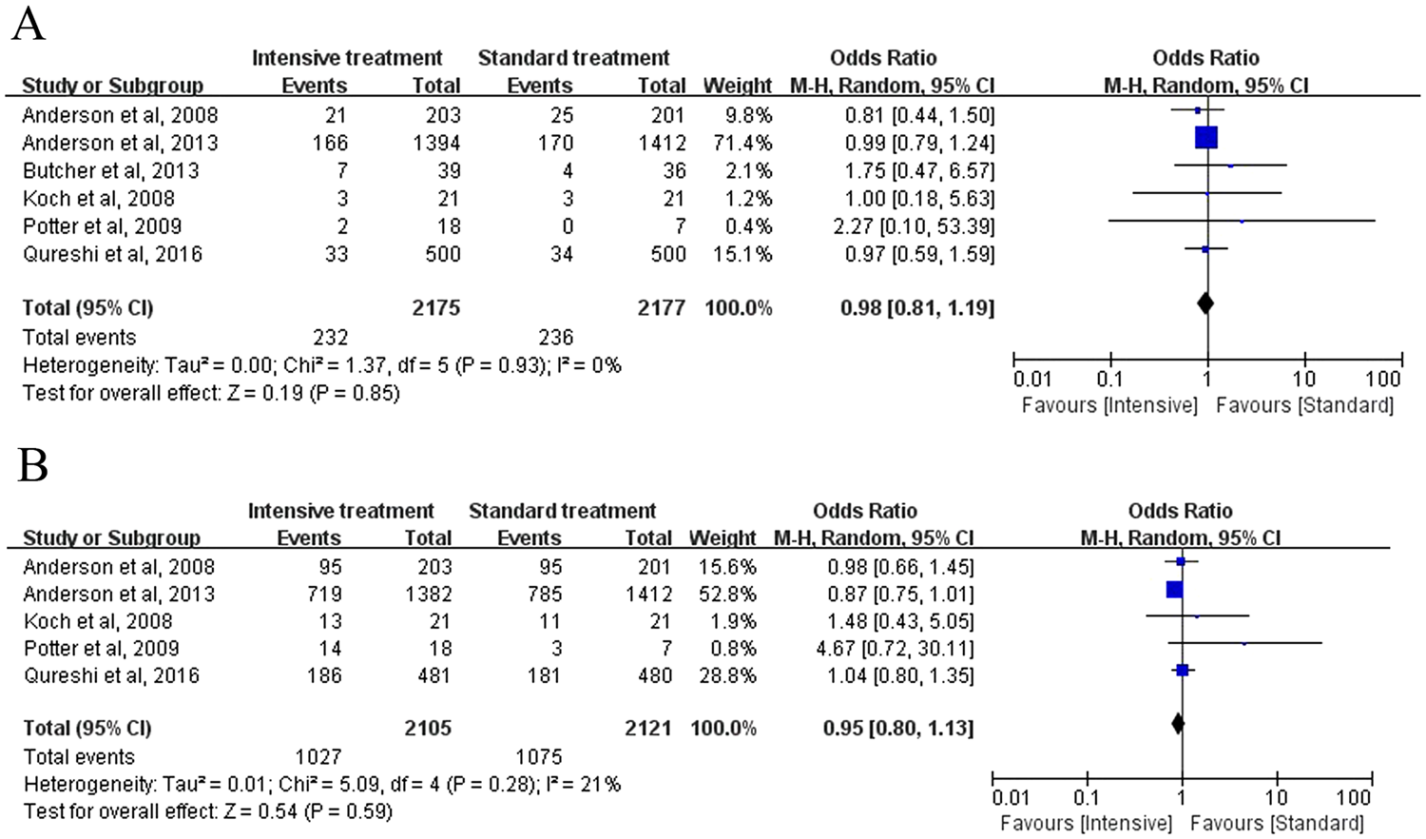
Forest plot of the comparison between intensive treatment and standard treatment: Primary outcomes: (**A**) Mortality; (**B**) Unfavorable outcomes.

The mortality rates had been reported in all 6 studies. After testing for heterogeneity, there was no statistical heterogeneity among these 6 trials (Chi-square = 1.37, *I* ^2^ = 0%, *P* = 0.93). Our findings indicated that intensive treatment was not associated with a reduction in the mortality rate compared with standard treatment (*P* = 0.85). The Odds ratio (OR) values in these 6 trials ranged from 0.81 to 2.27, with an overall OR of 0.98 (95% CI: 0.81–1.19; Fig. 3A).

#### Unfavorable outcomes

Five studies with 3,300 patients analyzed the unfavorable outcomes between the intensive and standard treatment groups. The heterogeneity among these 5 studies was low (Chi-square = 5.09, *I* ^2^ = 21%, *P* = 0.28). Pooled OR of unfavorable outcomes was 0.95 (95% CI: 0.80–1.13) and the incidence of unfavorable outcomes was not statistically significantly different between the intensive and standard treatment groups (*P* = 0.59; Fig. 3B).

### Secondary outcomes

#### Hematoma expansion at 2 hours

**Figure 4:**
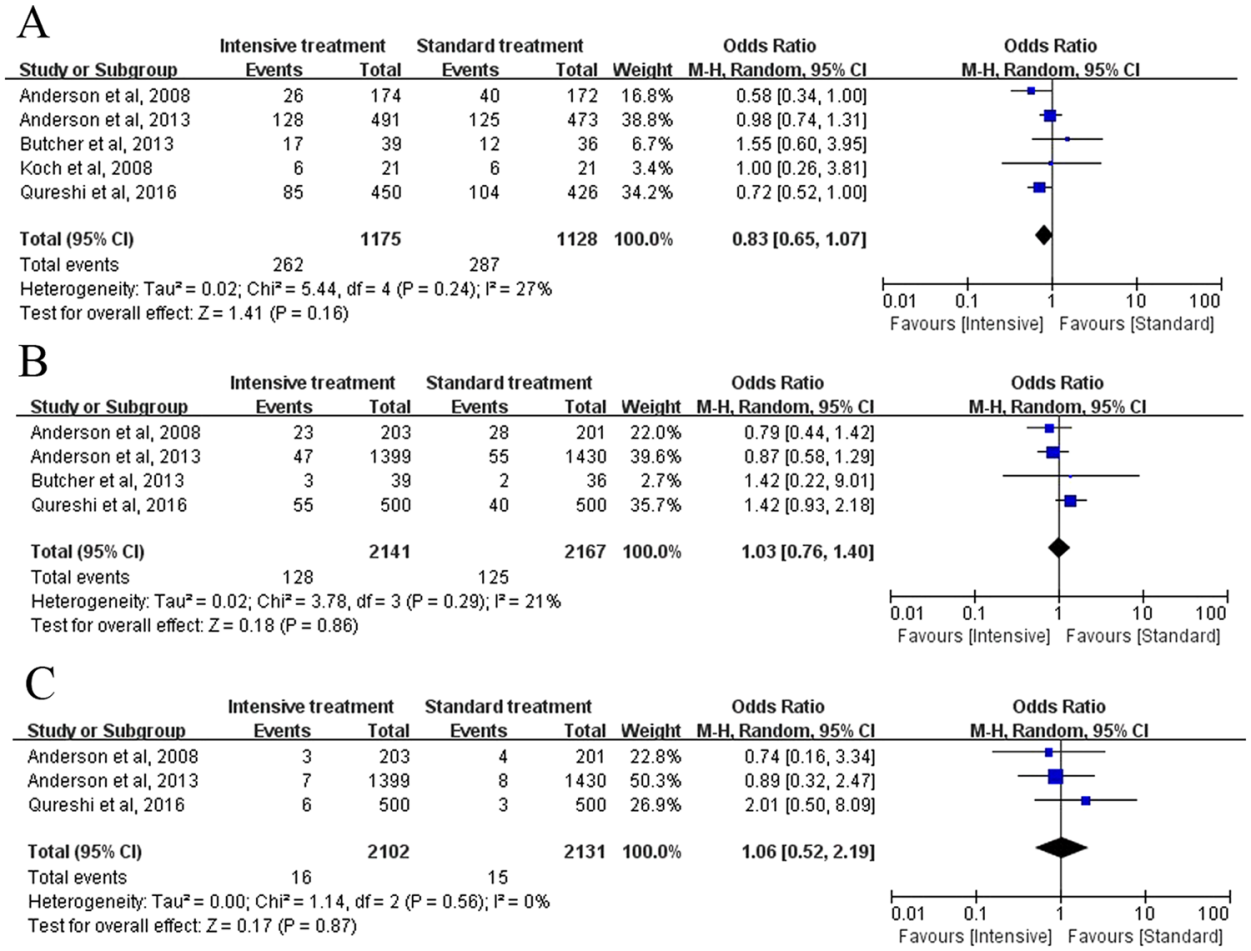
Forest plot of the comparison between intensive treatment and standard treatment: Secondary outcomes: (**A**) Hematoma expansion; (**B**) Neurologic deterioration; (**C**) Severe hypotension.

Five studies with 2,852 patients discussed hematoma expansion (HE) at 24 hours. Heterogeneity among these trials was low (Chi-square = 5.44, *I* ^2^ = 27%, *P* = 0.24). The overall OR was 0.83 (95% CI: 0.65–1.07), showing that the incidence of HE was not significantly different in the intensive vs standard treatment group (*P* = 0.16; Fig. 4A).

#### Neurologic deterioration

Four studies with 4,308 acute ICH patients compared the ratio of neurological deterioration. For the heterogeneity, *I* ^2^ value was 21% with *P* = 0.29. The pooled OR and 95% CI of neurological deterioration was 1.03 (95% CI: 0.76–1.40), showing that the difference between intensive treatment and standard treatment was not significant (*P* = 0.86; Fig. 4B).

#### Severe hypotension

Three studies with 4,233 patients were available for the analysis of severe hypotension. Heterogeneity among trials was low (Chi-square = 1.14, I^2^ = 0%, *P* = 0.56). The pooled OR was 1.08 (95% CI, 0.52 to 2.19), indicating no significant difference between two groups (*P* = 0.87; Fig. 4C).

### Pooled adverse event of BP reduction

We evaluated the occurrence of an adverse event (AE) associated with BP reduction. Events included: cardiovascular, renal, recurrent stroke, and non-cardiovascular events (Table 2). The risk ratio (RR) of each AE occurrence were greater than 1.0 with heterogeneity of *I* ^2^ < 30%. However, meta-analysis showed significant difference between two treatment approaches only for renal AE (*P* = 0.001). This indicated that the intensive treatment for acute ICH patients had higher risk of renal adverse effects than standard treatment. The *P*-values for the remaining RR were >0.05.

**Table 2:** Pooled risk ratios of adverse effects of blood pressure reduction in included studies.

Adverse effects(AE)	Number of studies	Number of patients Intensive/Standard	Risk ratio (RR) and 95%CI	*P*-value	I^2^ (%)
cardiovascular AE	3	86 / 77	1.09 [0.72, 1.66]	0.67	28
renal AE	2	49 / 22	2.34 [1.39, 3.92]	0.001	0
recurrent stroke	3	50 / 48	1.05 [0.69, 1.58]	0.83	0
non-cardiovascular AE	2	177 / 173	1.05 [0.84, 1.31]	0.68	0

### Subgroup analysis of unfavorable outcomes

We compared the unfavorable outcomes of various ICH subgroups based on age, history of hypertension, time from onset to randomization, baseline systolic BP, and National Institute of Health stroke scale (NIHSS) score at baseline.

#### Age

**Figure 5:**
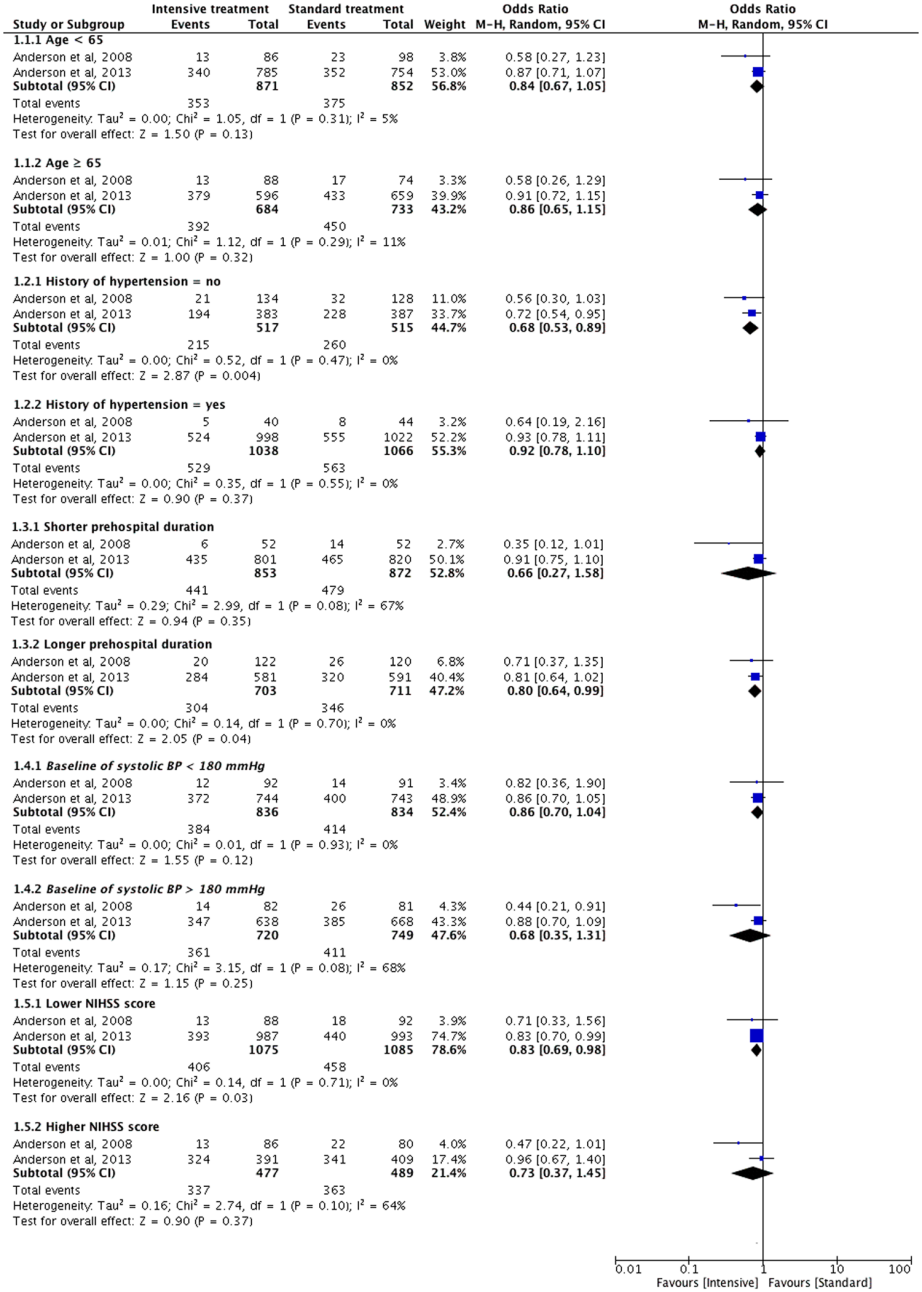
Forest plot of the subgroup analysis of unfavorable outcomes compared between intensive treatment and standard treatment.

Data from 2 studies was stratified and analyzed based on different age stages (≤65 years old subgroup, and >65 years old subgroup). There was no significant difference on unfavorable outcomes for the age ≤65-year-old subgroup (OR = 0.84, 95% CI: 0.67–1.05, *P* = 0.13); nor for the age >65-year-old subgroup (OR = 0.86, 95% CI: 0.65–1.15, *P* = 0.32) (Fig. 5).

#### History of hypertension

We stratified subgroups by hypertension history (Fig. 5). In patients with a history of hypertension, the summary OR was 0.92 with *P* of 0.37. However, in patients without a history of hypertension, the incidence of unfavorable outcomes in intensive treatment group was significantly lower than standard treatment group with a pooled OR of 0.68 (95% CI: 0.53–0.89, *P* = 0.004). These data suggest that the intensive treatment approach was a prior selection in acute ICH patients without hypertension history.

#### Time from onset to randomization

The time periods from onset to randomization were defined as shorter prehospital duration subgroup and longer prehospital duration subgroup according to the original data in included studies. In the shorter prehospital subgroup, overall OR value was not significant (OR = 0.66, P = 0.35). In the longer prehospital duration subgroup, the incidence of unfavorable outcomes in intensive treatment group was significantly lower than standard treatment group with a pooled OR of 0.80 (95% CI: 0.64–0.99, *P* = 0.04; Fig. 5).

#### Baseline of systolic BP

Baseline systolic BP was divided into two subgroups based on the level of 180 mm Hg. Neutral results (P > 0.05) were found for both subgroups analysis (Fig. 5). Hence, it appears to be unnecessary to take baseline systolic BP into account when selecting a treatment schedule.

#### NIHSS score at baseline

Subgroup analysis was stratified by NIHSS score (lower NIHSS score subgroup and higher NIHSS score subgroup). In the lower NIHSS score subgroup, the pooled OR value was 0.83 (*P* = 0.03), suggesting the significantly lower incidence of unfavorable outcomes in intensive treatment group than in standard treatment group. In the higher NIHSS score subgroup, we didn’t find any significant difference (Fig. 5).

## Discussion

The present meta-analysis of 6 RCTs and 4,385 acute ICH patients explored the influence of systolic BP reduction on acute ICH outcomes. Our results demonstrated that the differences of primary outcomes (mortality and unfavorable outcomes) and secondary outcomes (HE at 24 hrs, neurologic deterioration, severe hypotension) were not significant between the intensive treatment group and standard treatment group. Moreover, the risk of renal AE in intensive treatment was significantly higher compared with standard treatment. Most importantly, the subgroup analysis found that the acute ICH patients treated using an intensive approach had lower rate of unfavorable outcomes for patients with no history of hypertension, or longer prehospital duration, or lower NIHSS score.

There were four meta-analyses published previously^14–16, 19^. Of these, only one meta-analysis that included four RCTs found a significant association between intensive BP lowering and reduction of hemorrhage expansion associated with improved clinical functional outcome^15^. The other 3 meta-analyses were consistent with our findings in that they detected no significant relationships between intensive treatment and poor clinical outcomes^14, 16, 19^. Notably, we found for the first time that selected patients with no history of hypertension, or longer prehospital duration, or lower NIHSS score might benefit from intensive BP lowering therapy.

Most of the observational studies found a tight association between BP and HE in patients with acute ICH^20, 21^. Improving clinical outcomes with intensive BP reduction treatment was hypothesized to be related to reduce HE^22^. It was speculated that intensive BP reduction improved outcomes through attenuation of absolute hematoma volume increasing at 24 hrs before and after adjustment for potential confounders^15, 23^. In addition, researchers found intensive BP reduction did not increase the volume of critically hypoperfused border-zone or perihematoma tissue, which supporting the safety of intensive BP reduction in acute ICH^24^. However, other studies did not find an association between BP and HE within the first 24 hrs^14, 25^. Therefore, as previously demonstrated, there remained doubtful as to the association of benefit from intensive treatment of BP for clinical outcomes in ICH.

Research has indicated that renal dysfunction is associated with intensive BP reduction after acute ICH^26, 27^. In a randomized pilot trial (INTERACT), renal failure was reported in 2% of acute ICH patients following intensive BP reduction^11^. Qureshi *et al*.^9^ reported that the rate of renal AE within 7 days was significantly lower in the standard treatment than intensive treatment. Consistent with the previous results, our meta-analysis results showed the pooled renal AE associated with BP reduction in intensive treatment had higher RR. As patients with renal failure have significantly worse outcomes in acute ICH, intensive BP lowering provides similar treatment effects irrespective of degree of renal failure^28^. Given that because the kidney contains many small vessels; severe hypertension usually leads to renal arteriolar sclerosis and hypotension also causes renal blood perfusion inadequacy and then renal failure. So, we speculate that intensive BP lowering contributes to higher RR of renal AE because of the hypertension before treatment and subsequent hypotension after treatment. Therefore, it is essential to carefully monitor the renal function in reducing systolic BP for acute ICH patients, especially for those with a history of hypertension.

In our subgroup analysis, results showed that the acute ICH patients treated by intensive approach had lower rate of unfavorable outcomes in subgroups of with no history of hypertension, or longer prehospital duration, or lower NIHSS score. These findings were the first to reported and might play an important role in choosing an appropriate therapeutic approach for selected patients. However, Anderson *et al*.^10^ found no significant effect of hypertension history on the effectiveness of intensive treatment. The possible reason was that patients with hypertension had an upward shift in cerebral autoregulation and possibly underwent increased risk of cerebral ischemia related to intensive BP lowering^29^. In the present study, subgroup analysis of onset age and the systolic BP at baseline of acute ICH patients was not significant. However, the INTERACT2 cohort^30^ detected that older people had more severe acute ICH status and worse outcomes (mortality, disability and quality of life). Moreover, the subgroup analysis of time from onset to randomization and NIHSS score baseline indicated that selected patients with longer prehospital duration or lower baseline NIHSS score might benefit from intensive treatment. This might be due to that longer prehospital duration led to progressive deterioration of HE, which would be more sensitive to intensive BP lowering. In contrast, patients with shorter prehospital duration might differ in the degree of damage. Therefore, the effectiveness of standard treatment and intensive treatment could not be differentiated, and need to be identified by further study with similar brain injury. Patients with lower NIHSS score might suffer mild brain damage, and thus benefit much from intensive BP lowering by saving more perihematoma tissue. While patients with higher NIHSS would suffer severe brain damage and thus BP lowering might be not helpful, for whom surgery would be an alternative treatment.

There are several limitations to this analysis. First, in any meta-analysis, publication bias is always considered as a major potential threat to validity^31^. We minimized the risk of publication bias through our extensive and careful retrieval. Second, we included 3 RCTs with relative small samples and low quality. Third, in our subgroup analysis, we included only 2 RCTs for each subgroup, which reduced the statistical power to detect a difference. Fourth, the different research outcomes may be influenced by either observer bias or selection bias, even though three authors (S.G., C.L., D.F.Z.) selected papers with strict inclusion criteria and exclusion criteria, the bias had more or less effect on the results and conclusion. Hence, high quality and large sample size RCTs are needed to confirm these results.

## Methods

### Research protocol

This study was performed according to the preferred reporting items for systematic reviews and meta-analyses (PRISMA) guidelines^32^, and the protocol of this meta-analysis has not been previously registered.

### Types of outcome measurements

We evaluated the following outcomes: a) mortality rates; b) unfavorable outcomes (defined as a modified Rankin Scale score of 4–6); c) hematoma expansion; d) neurologic deterioration (defined as an increase from baseline to 24 hours of 4 or more points on the NIHSS or a decrease of 2 or more points on the Glasgow Coma Scale); e) severe hypotension; f) adverse events.

### Inclusion and exclusion criteria

Studies were included if they were RCTs meeting the following criteria: a) including patients (more than 18 years old and both genders) with radiologically confirmed ICH; b) randomizing patients to either the intensive BP lowering treatment group or standard BP lowering treatment group; c) measuring the outcomes we listed above; d) being sufficient to allow calculation of effect sizes.

Studies were excluded if they included one of the following circumstances: a) patients who had serious cardiovascular diseases, cancers, and other visceral functional diseases; b) age of participants were less than 18 years old; c) studies without the previous definition of outcomes or the variables were different from our measurements.

### Literature search strategies

We conducted a search of sufficient rigor in the literature library of: Embase, PubMed, Scopus, Cochrane library, and Essential Evidence from 1980 to August 2016. The search included a combination of the free words ‘intracranial hemorrhage’ and ‘blood pressure’, or the key words ‘intracerebral hemorrhage’ and ‘blood pressure’. No language or other restrictions were imposed. The search strategies were showed in detail in Fig. 1. We have checked reference lists of all articles that met the criteria and examined relevant review articles to identify studies that may have been missed in the database search.

### Study selection and data collection process

Three authors (S.G., C.L. and D.F.Z.) independently searched and read the titles, abstracts and full text of literatures obtained from electronic databases and excluded studies that were irrelevant to our object. The authors discussed and resolved the disputes together. Z Li, R Chen and P Sheng extracted the following data from included studies independently: the name of first author, publication year and country of each literature, the sample size of intensive and standard treatment group, ratio of sex, mean age, duration of follow-up (months), outcomes, baseline and target BP level of both groups (Table 1).

### Statistical analysis and quality assessment

We conducted this meta-analysis using Review Manager version 5.2 software (http://tech.cochrane.org/revman). ORs and RRs were used to express the comparison between intensive BP reduction and standard BP reduction in acute ICH patients. Heterogeneity among studies was assessed by the *I* ^2^ statistics. Higher values of *I* ^2^ indicated greater degree of heterogeneity. The value of *I* ^2^ ≤ 50% denoted acceptable heterogeneity among studies, with *I* ^2^ > 50% were considered to suggest substantial heterogeneity, while values *I* ^2^ ≥ 75% indicated considerable heterogeneity^33, 34^. Random-effect model was used in data synthesis process. In order to maximize the power of the test, Egger test was conducted to assess the publication bias. Risk of bias of included studies were assessed based on the recommendation of Cochrane Collaboration, including selection bias, performance bias, detection bias, attrition bias and reporting bias.
